# Breaking the Cycle of Inactivity: The Impact of Exercise on Pain, Distress, and Functionality in Knee Osteoarthritis

**DOI:** 10.1093/geroni/igaf122.2148

**Published:** 2025-12-31

**Authors:** Donya Nemati, Jacob Jahn, Levi Travis, Ryan Rizk, Eric Kholodovsky, Navin Kaushal, Thomas Best

**Affiliations:** Ohio State University, Columbus, Ohio, United States; University of Miami Miller School of Medicine, Miami, Florida, United States; University of Miami Miller School of Medicine, Miami, Florida, United States; University of Miami Miller School of Medicine, Miami, Florida, United States; University of Miami, Coral Gables, Florida, United States; Indiana University, Indianapolis, Indiana, United States; University of Miami, Coral Gables, Florida, United States

## Abstract

**Introduction:**

Knee osteoarthritis (KOA) is a chronic condition marked by joint degeneration that leads to pain, functional limitations, and diminished quality of life. The cyclical nature of KOA symptoms creates a self-perpetuating loop: reduced physical activity raises body mass index (BMI), increasing joint stress and worsening symptoms, further discouraging exercise. The purpose was to investigate the relationship between clinical measures of KOA and physical activity-related variables.

**Methods:**

Patients (n = 704; mean age 56.42) with symptomatic KOA (K-L grades 0–4) were evaluated at the University of Miami’s sports medicine clinic from 2020-2024. Clinical measures of pain, psychological distress, and functional limitations were assessed using the VAS, PCS, and WOMAC. Validated exercise variables included Physical Activity (PA), Social Support (SS), Exercise Intention (EI), and Exercise Self-Efficacy (ESE). Multivariable linear regression assessed the relationship between clinical and exercise measures, while ANOVA was used for gender comparisons in exercise-related measures.

**Results:**

PA was associated with VAS (β=-0.037, p<.001), PCS scores (β=-0.124, p<.001), and WOMAC scores (β=-0.364, p<.001). SS for exercise was associated with lower VAS (β=-0.017, p = 0.042) and WOMAC scores (β=-0.203, p<.001). ESE was associated with reduced PCS (β=-0.061, p = 0.017) and WOMAC scores (β=-0.267, p<.001). Gender comparisons revealed that males scored higher than females in EI: F(1, 734)=4.69, (p = 0.031), ESE: F(1, 733)=7.36, (p = 0.007), PA: F(1, 289)=6.03, (p = 0.015), and SS F(1, 734)=4.32, p = 0.038.

**Conclusion:**

Higher PA significantly reduced pain and functional limitations in KOA patients. Gender discrepancies in self-efficacy and intention to exercise highlight the need for exercise interventions tailored for males and females.

